# Differential Regulation of Cardiac Function and Intracardiac Cytokines by Rapamycin in Healthy and Diabetic Rats

**DOI:** 10.1155/2017/5724046

**Published:** 2017-03-20

**Authors:** Christian Luck, Vincent G. DeMarco, Abuzar Mahmood, Madhavi P. Gavini, Lakshmi Pulakat

**Affiliations:** ^1^Department of Medicine, University of Missouri, Columbia, MO, USA; ^2^Harry S. Truman Memorial Veterans Affairs Hospital, Columbia, MO, USA; ^3^Novopyxis, Boston, MA, USA; ^4^Department of Nutrition and Exercise Physiology, University of Missouri, Columbia, MO, USA

## Abstract

Diabetes is comorbid with cardiovascular disease and impaired immunity. Rapamycin improves cardiac functions and extends lifespan by inhibiting the mechanistic target of rapamycin complex 1 (mTORC1). However, in diabetic murine models, Rapamycin elevates hyperglycemia and reduces longevity. Since Rapamycin is an immunosuppressant, we examined whether Rapamycin (750 *μ*g/kg/day) modulates intracardiac cytokines, which affect the cardiac immune response, and cardiac function in male lean (ZL) and diabetic obese Zucker (ZO) rats. Rapamycin suppressed levels of fasting triglycerides, insulin, and uric acid in ZO but increased glucose. Although Rapamycin improved multiple diastolic parameters (*E*/*E*′,* E*′/*A*′,* E*/*Vp*) initially, these improvements were reversed or absent in ZO at the end of treatment, despite suppression of cardiac fibrosis and phosphoSer473Akt. Intracardiac cytokine protein profiling and Ingenuity® Pathway Analysis indicated suppression of intracardiac immune defense in ZO, in response to Rapamycin treatment in both ZO and ZL. Rapamycin increased fibrosis in ZL without increasing phosphoSer473Akt and differentially modulated anti-fibrotic IL-10, IFN*γ*, and GM-CSF in ZL and ZO. Therefore, fundamental difference in intracardiac host defense between diabetic ZO and healthy ZL, combined with differential regulation of intracardiac cytokines by Rapamycin in ZO and ZL hearts, underlies differential cardiac outcomes of Rapamycin treatment in health and diabetes.

## 1. Introduction

Accumulating evidence supports the role of the mechanistic (previously called mammalian) target of rapamycin (mTOR) as the “Grand Bioconductor” of metabolism and aging [1–3]. The mTOR protein kinase serves as a central integrator of nutrient signaling pathways and is overactivated in cardiovascular tissues of patients with metabolic disorders such as obesity, type 2 diabetes (T2DM), and metabolic syndrome (MetS) [4–8]. Overactivation of mTOR is linked to cardiovascular disease. We have reported recently that inhibition of mTOR complex 1 (mTORC1) by Rapamycin in male Zucker obese (ZO) rat results in weight loss and reduction in body fat and lean mass [9]. mTORC1 consists of mTOR, regulatory-associated protein of mTOR (Raptor); mammalian lethal with Sec13 protein 8 (mLST8, also known as GbL); proline-rich Akt substrate 40 kDa (PRAS40); and DEP-domain-containing mTOR-interacting protein (Deptor) [4, 5]. Rapamycin suppresses mTORC1 pathway by inhibiting phosphorylation of p70S6 kinase (p70S6K) at Thr389, which in turn no longer activates ribosomal protein S6 and protein synthesis machinery. We also demonstrated that 12 weeks of Rapamycin treatment in ZO rats resulted in downregulation of cardiac levels of microRNA miR-208a. miR-208a is a marker for cardiac dysfunction and fibrosis. 12 weeks of Rapamycin treatment also increased expression of cardiac Mediator Complex 13 (MED13) which is known to improve whole body metabolism [9]. Rapamycin is an FDA-approved immunosuppressant and anticancer agent that inhibits mTOR protein kinase [4, 5, 10]. Rapamycin is now considered to be an antiaging agent and recent reports suggest that Rapamycin treatment increases lifespan in mice [11, 12].

Obesity and T2DM are metabolic disorders characterized by chronic inflammation and accelerated aging. They, in turn, further exacerbate cardiovascular disease (CVD) [13]. Recent studies on db/db mice treated with Rapamycin for 4 weeks have shown that Rapamycin could improve cardiac contractile functions [14]. Moreover, in isolated db/db mice hearts, Rapamycin treatment reduced oxidative stress and protected against reperfusion injury [15]. Therefore, short-term treatment with Rapamycin is cardioprotective in T2DM. However, long-term treatment of db/db mice with Rapamycin increased their mortality rate [16]. Thus, the protective effect of Rapamycin seems to depend on the length of treatment and the metabolic health of the subject (healthy or diabetic). There are no reports of chronic Rapamycin treatment causing rebound elevation of mTORC1. However, activation of Rapamycin-insensitive mTORC2 is implicated in some of these effects [4, 5]. Understanding how the protective effects of Rapamycin in cardiac tissues are modulated by the metabolic and cardiac microenvironment induced in T2DM compared to healthy subjects is an important aspect to ensure effective use of this drug for cardioprotection in patients with preexisting conditions including diabetes.

As an immunosuppressant, Rapamycin is expected to modulate circulating and systemic cytokine profiles. The extent to which Rapamycin modulates intracardiac cytokine profiles differently in diabetes compared to healthy patients is not fully understood. We observed that Rapamycin (750 *μ*g/kg/day; 12 weeks) reduced body weight, body fat, and cardiac microRNA miR-208a (implicated in promoting hypertrophy and fibrosis) and increased cardiac Med13 (that promotes whole body metabolism in Zucker diabetic fatty rats) [9]. Based on these observations, we hypothesized that Rapamycin treatment would mitigate diastolic dysfunction in these rats. We also hypothesized that Rapamycin would improve their intracardiac cytokine profile to mitigate chronic inflammation. Therefore, we evaluated cardiac parameters of healthy Zucker lean (ZL) rats and diabetic Zucker obese (ZO) rats after six-week and 12-week Rapamycin treatments (ZL-R and ZO-R). We also investigated changes in the intracardiac cytokine protein profiles of these rats at the end of 12-week Rapamycin treatment.

## 2. Methods

### 2.1. Rapamycin Treatment of Rats

All animal procedures used in this study were approved by the Harry S. Truman Veterans Memorial Hospital (HSTVMH) Subcommittee for Animal Safety and University of Missouri IACUC before commencing. All animals were cared for in accordance with the Guidelines for the Care and Use of Laboratory Animals (National Institutes of Health publication 85-23). Zucker obese (fa/fa) (ZO) and lean (ZL) rats (Charles River Laboratories) were used in this study. Rats were maintained on ad libitum food and water and housed singly at the HSTVMH animal housing facility under standard laboratory conditions. Room temperature was maintained at 21-22°C. Light and dark cycles were for 12 hours, but animals were entrained to have dark cycle (awake time) during the day and light cycle (sleep time) during the night. At 8 weeks of age, Rapamycin pellets designed to deliver Rap at a concentration of 750 *μ*g/kg/day for 21 days (from Innovative Research of America Inc., Sarasota, FL) or placebo (sugar) pellets were placed surgically under the skin behind the shoulder blades under brief isoflurane anesthesia and this procedure was repeated 3 times to achieve a 12-week treatment. ZL and ZO rats that received placebo pellets are referred to as ZL-C and ZO-C and those implanted with Rapamycin pellets are referred to as ZL-Rap and ZO-Rap, respectively, in the text.

### 2.2. Fasting Plasma Profile and Tissue Collection

Animals were fasted for 6 hours before blood collection. Blood was collected biweekly from the saphenous vein as described previously [17]. Blood was also collected by cardiac puncture at the time of sacrifice according to IACUC approved procedure as described previously [17]. Plasma analysis was performed by Comparative Clinical Pathology Services at Columbia, MO. Plasma levels of triglycerides and uric acid were measured using commercially available assays (Beckman-Coulter, Brea, CA) on an automated clinical chemistry instrument (AU680, Beckman-Coulter, Brea, CA). Glucose and insulin were measured by an automated hexokinase G-6-PDH assay and an ELISA kit specific for rat insulin, respectively. Hearts were harvested at time of sacrifice as described before [17], flash frozen in liquid nitrogen, and stored at −80°C for future use.

### 2.3. Echocardiography

Transthoracic echocardiography on placebo or Rapamycin-treated rats was performed under inhaled isofluorane anesthesia (0.5–1.0% maintenance) with a 12 MHz pediatric transducer using a GE Vivid I Ultrasound system to assess in vivo cardiac morphology and diastolic and systolic function as previously described [8]. Echocardiography was performed in the middle of treatment (after six weeks) and at the end of treatment (after 12 weeks).

### 2.4. Histopathology and Interstitial Fibrosis Measurement

Tissues from animals (randomly numbered) were fixed in 10% neutral buffered formalin (NBF), embedded into paraffin blocks; sections were cut at 4 *μ*m thickness and were stained with Masson's Trichrome Stain (MTS) at Research Animal Diagnostic Laboratory (RADIL), Columbia, MO. The stained sections were scanned using the Aperio CS Slide Scanner by WSI Analytics Lab, Department of Pathology and Anatomical Sciences, University of Missouri, Columbia, MO. Scanned sections were visualized using Aperio ImageScope (Leica Biosystems). Next, 10 interstitial (20x magnification) images of the most fibrotic regions were selected per animal. Fibrotic area was quantified using the in-built Positive Pixel Count (V9) algorithm (settings were manually determined as follows: hue value = 0.6785; hue width = 0.4; color saturation threshold = 0). Positivity (Positive/Total Pixels) was averaged over all regions from a single group to determine mean fibrotic area per group.

### 2.5. Analysis of Rat Intracardiac Cytokine Protein Profile by Quantibody® Rat Cytokine Array 67

Frozen heart tissues (−80°C) of placebo- or Rapamycin-treated rats (*n* = 5 per group) were powdered under liquid nitrogen and lysed with ice cold lysis buffer (Cat#: AA-Lys, RayBiotech, Norcross, GA) supplemented with Okadaic Acid (0.1 *μ*M), sodium orthovanadate (250 *μ*M), sodium pyrophosphate (650 *μ*M), Bestatin (5 nM), and 1x cOmplete™ Protease Inhibitor Cocktail (Roche Life Sciences). Tissue lysis was performed using a Qiagen TissueLyser. Cell debris was removed by centrifugation and protein in the supernatant was estimated by BCA method (Pierce BCA protein assay kit). Intracardiac cytokine protein levels of randomly numbered animals were analyzed using RayBiotech's Quantibody Rat Cytokine Array 67 [18, 19]. This array is a combination of 2 nonoverlapping arrays that facilitates quantitative measurement of the concentrations of 67 rat cytokines by using appropriate antibody pairs. Within the array, each individual cytokine was represented four times, along with positive and negative controls which allowed for an analysis of standard deviation. Cytokine analysis of the heart tissue lysates was performed by RayBiotech according to their protocol and software analysis. Cytokines that exhibited statistically significant differences (*p* < 0.05) between different groups were selected for input into Ingenuity Pathway Analysis (IPA, Qiagen, Germantown, MD) to identify diseases and functions that were affected. Heatmaps were generated using the ggplot2 package for R [20].

### 2.6. Western Blotting

To determine the changes in the phosphorylation status of Ser473 of Akt in response to Rapamycin treatment of ZL and ZO rats, their cardiac tissue lysates were subjected to Western blotting as described previously [9, 17]. Akt and pSer473Akt antibody were purchased from Cell Signaling Technology, Danvers, MA. Tris-buffered saline-Tween 20 (TBST) containing 5% bovine serum albumin (BSA) was used for blocking the Western blots (PVDF) for one hour. Primary antibodies were diluted 1 : 1000 in 5% BSA in TBST. Blots were incubated for overnight at 4°C in primary antibodies, were washed with TBST, and were incubated in the horseradish peroxidase-conjugated secondary antibody (1 : 25,000 dilution in 5% BSA in TBST). After TBST washes, chemiluminescent substrate (Supersignal West Femto Maximum Sensitivity Substrate kit; Thermo Scientific) was added to visualize antibody binding using Bio-Rad ChemiDoc XRS image-analysis system. Quantitation of pSer473 protein band density compared to total Akt protein band density was performed after normalizing density of each band to the density of total protein (determined by amido black staining) in different areas of its respective lane of the blot. All protein band density quantifications were performed using Quantity One software (Bio-Rad Laboratories Inc., Berkeley, Ca). Data are reported as the normalized protein band density in arbitrary units.

### 2.7. Statistical Analysis

Results are reported as means ± SE. Statistical analysis was performed using SigmaStat software. For multiple comparisons, one- or two-way ANOVA or two-way repeated measures ANOVA, followed by uncorrected Fisher's LSD, was performed as appropriate, with main effects of strain, treatment, or a strain *∗* treatment interaction (INT) noted where relevant. Unpaired two-tailed* t*-test was performed for pairwise comparisons. A *p* value < 0.05 was deemed significant.

## 3. Results

### 3.1. Rapamycin Treatment Reduced Fasting Plasma Insulin, Triglycerides, and Uric Acid

**Figure 1:**
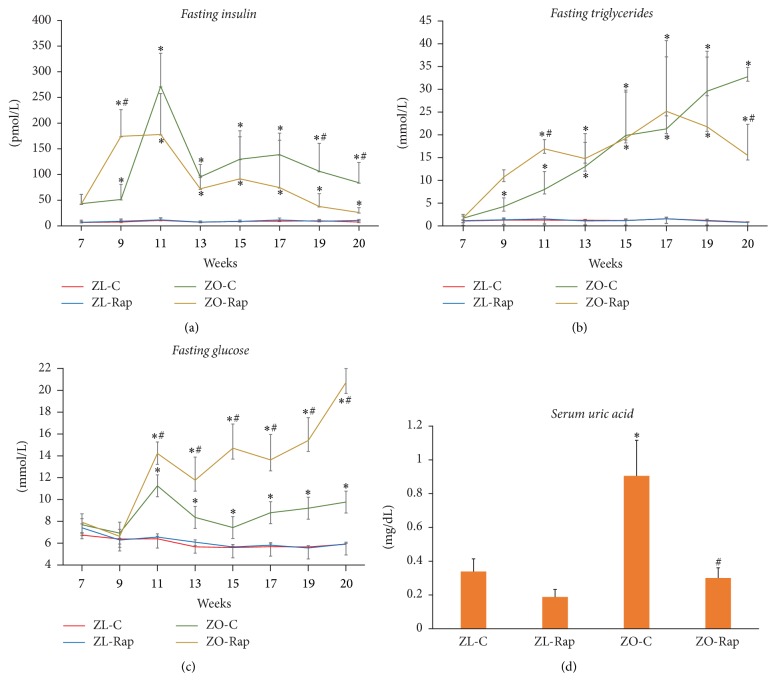
*Changes in fasting serum triglyceride levels, insulin, and glucose, and serum uric acid of lean and obese rats treated with and without Rapamycin*. Six-hour fasting blood collection of the indicated groups was performed for analysis of (a) serum insulin, (b) serum triglyceride levels, and (c) plasma glucose. (d) Uric acid was measured from serum samples collected at the time of sacrifice. Analysis was performed using commercially available assays (Beckman-Coulter, Brea, CA) on an automated clinical chemistry instrument (AU680, Beckman-Coulter, Brea, CA) for triglycerides, glucose, and uric acid. Insulin was measured by an ELISA kit specific for rat insulin. Values are means ± SEM. *n* = 6 for ZL-C, ZL-Rap, and ZO-C, and *n* = 5 for ZO-Rap. ^*∗*^*p* < 0.05 versus ZL-C, and ^#^*p* < 0.05 versus ZO-C using two-way repeated measures ANOVA or Student's* t*-test as appropriate.

We have reported previously that Rapamycin treatment (750 *μ*g/kg/day) of ZO rats for a period of 12 weeks significantly suppressed their food intake, body weight, body fat, and lean muscle mass [9]. Here we report that, starting around 8 weeks of age, ZO-C exhibited significantly high levels of fasting plasma insulin and triglycerides (Figures 1(a) and 1(b)). Starting at around 9 weeks of age, they also showed high levels of fasting plasma glucose (Figure 1(b)). Rapamycin suppressed fasting plasma insulin and triglycerides but elevated fasting plasma glucose in ZO rats (Figure 1). At the end of the treatment, we observed an increase in fasting plasma glucose (over 2-fold higher) in ZO-Rap compared to ZO-C (Figure 1(c)). Conversely, plasma insulin levels were suppressed by about 50% in ZO-Rap compared to ZO-C (Figure 1(a)). At the end of treatment, serum from ZO-C had high levels of uric acid, a marker of chronic inflammation and heart failure [21] compared to ZL-C. Rap treatment suppressed uric acid elevation (Figure 1(d)). Rapamycin treatment did not change these parameters significantly in ZL rats (Figure 1).

### 3.2. Effect of Rapamycin on Cardiac Functional Parameters in ZO and ZL Rats after 6- and 12-Week Treatments

At the end of 6-week Rapamycin treatment (14 weeks of age), the cardiac functional parameters of untreated and Rap-treated ZL and ZO rats were determined by echocardiography. Fourteen-week-old ZL-C and ZL-Rap rats exhibited similarly normal ultrasound-derived cardiac structural and functional parameters (Table 1). Compared to ZL-C, ZO-C rats exhibited abnormalities in multiple diastolic parameters, including decreases in the tissue doppler* E*′/*A*′ ratio indicative of impaired septal wall motion and mitral inflow propagation velocity (*Vp*) indicative of impaired active phase relaxation. They also showed increases in LV filling pressure (*E*/*E*′ and* E*/*Vp* ratios), isovolumic relaxation time (IVRT), and myocardial performance index (Tei index of global cardiac function) (Table 1). However, ejection fraction (EF) and fractional shortening (FS) were similar in ZL and ZO groups with and without Rapamycin treatment (Table 1). Collectively, these indicate that the ZO rats exhibited diastolic dysfunction with preserved ejection fraction. These observations are consistent with cardiac pathology in diabetic rats [22]. With the exception of IVRT which was similarly prolonged in both ZO groups, these diastolic abnormalities were improved in ZO rats treated for 6 weeks with Rap (Table 1).

**Figure 2:**
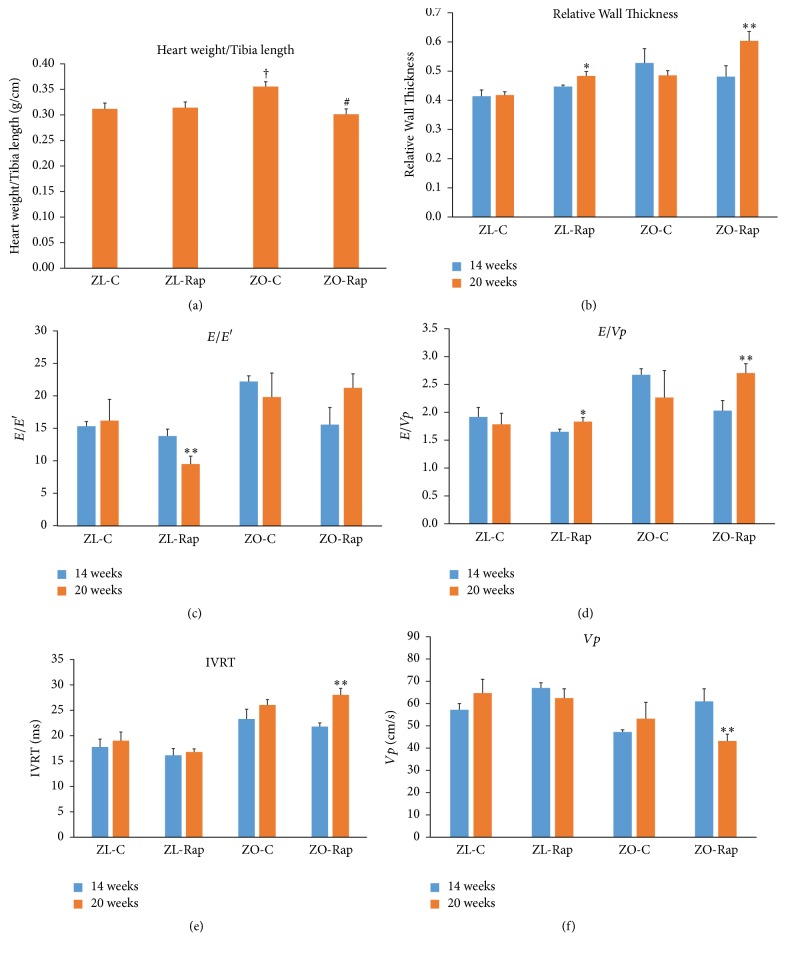
*Heart weight, cardiac function, and myocardial strain analysis in 14- and 20-week-old rats*. (a) Graph shows heart weight determined at the time of sacrifice after normalizing to tibial length. (b) LV relative wall thickness (RWT) calculated by using the formula PWTd + AWd/LVIDd, where AW is the anterior LV diastolic wall thickness and LVID is the LV internal diameter. (c) Graph shows* E*/*E*′, a powerful predictor of primary cardiac event in humans, (d)* E*/*Vp*, (e) isovolumic relaxation time (IVRT), (f) flow propagation velocity (*Vp*). Values are means ± SEM. *n* = 6 for ZL-C, ZL-Rap, and ZO-C, and *n* = 5 for ZO-Rap for (a) ^†^*p* < 0.05 versus ZL-C, ^#^*p* < 0.05 versus ZO-C. For (b)–(f), *n* = 4 for all groups, ^*∗*^*p* < 0.1 and ^*∗∗*^*p* < 0.05 compared to 14 weeks for each respective group. *p* values were determined using two-way repeated measures ANOVA or Student's* t*-test as appropriate.

At the end of the 12-week Rapamycin treatment, both ZL-C and ZL-Rap rats exhibited normal ultrasound-derived cardiac structural and functional parameters (Table 2). A notable exception was the increase in the* E*′/*A*′ ratio in ZL-Rap, and this was due mainly to an increase in septal wall velocity during early diastole (*E*′), indicative of improved diastolic filling (Table 2). Fractional shortening and ejection fraction were similar between the ZL and ZO groups with and without treatment (Table 2). Rapamycin reduced heart weight (adjusted to tibia length) in ZO rats (Figure 2(a)). There were significant strain effects in diastolic parameters indicating impaired diastolic dysfunction in the older group of ZO rats compared to age-matched ZL rats and this was due largely to impaired diastolic parameters in the ZO-Rap group (Table 2). Specifically, measures of filling pressure (*E*/*E*′ and* E*/*Vp*), isovolumetric relaxation time (IVRT) and propagation velocity (*Vp*) tended to be more impaired in the ZO-Rap rats that received Rapamycin treatment for 12 weeks compared to the earlier time point (Figures 2(b)–2(f)). Thus, after the six-week treatment, Rap improved cardiac parameters in ZO group, but, after the 12-week treatment, these improvements were reversed or not detectable.

### 3.3. Effect of Rapamycin Treatment on Cardiac Fibrosis and Phosphorylation Status of Ser473 Akt

**Figure 3:**
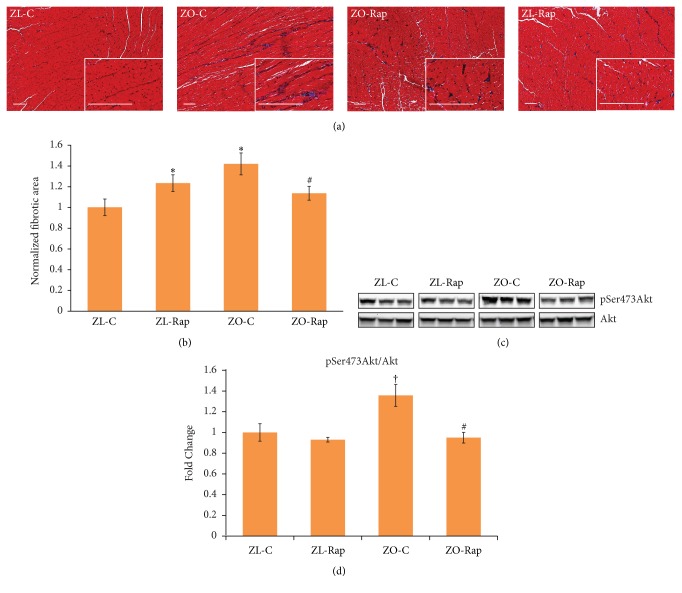
Effect of Rapamycin treatment on fibrosis and phosphorylation status Ser473 residue of Akt in ZL and ZO rats. (a) Representative images of Trichrome stained heart sections of ZL-C, ZL-Rap, ZO-C, and ZO-Rap are shown. Images were taken at 10x magnification and the insets were taken at 40x magnification (scale bars = 200 *μ*m). (b) Graph shows the cumulative data for normalized fibrotic area from 10 images for each animal (*n* = 5 animals per group; 50 images total for one group). Fibrosis was higher in the heart tissue sections of ZO-C and ZL-Rap compared to that of ZL-C. ZO-Rap heart tissue sections displayed reduced fibrosis compared to ZO-C. (c) Representative images of Western blots probed with anti-phosphoSer473 Akt and anti-Akt antibodies. Data for 3 different animals per group are shown. All bands are from the same gel. (d). Bar graph shows the ratio of intensity of bands corresponding to phosphoSer473 Akt to total Akt after normalizing to total protein levels (*n* ≥ 3). ^*∗*^*p* < 0.05 for ZL-C versus ZO-C or ZO-Rap and ^#^*p* < 0.05 for ZO-Rap versus ZO-C and ^†^*p* = 0.06 for ZO-C versus ZL-C.

We have reported previously that Rapamycin treatment suppressed miR-208a that promotes fibrosis in ZO rats [9]. To further confirm that Rapamycin treatment suppressed cardiac fibrosis, we determined the extent of cardiac fibrosis in all four groups. As shown in Figure 3, Rapamycin treatment suppressed interstitial fibrosis in ZO rats (Figures 3(a) and 3(b)). This is consistent with the effect of Rapamycin treatment according to previously published reports[14]. However, Rapamycin treatment increased cardiac fibrosis in ZL rats. Rapamycin is reported to activate Akt and activation of Akt caused by phosphorylation of Ser473 residue (phosphoSer473Akt) is known to promote cardiac fibrosis [23, 24]. To test whether Rapamycin-mediated activation of Akt plays a role in the increase in cardiac fibrosis in ZL rats, we examined the phosphorylation status of Ser473 of Akt. We found that Ser473 phosphorylation of Akt was increased (*p* = 0.06) in ZO-C heart compared to ZL-C heart and this effect is consistent with the increased cardiac fibrosis in ZO-C compared to ZL-C (Figures 3(c) and 3(d)). Moreover, Ser473 phosphorylation of Akt was suppressed by Rapamycin in ZO rats (*p* = 0.04) and this is consistent with the Rapamycin-induced suppression of cardiac fibrosis in ZO-Rap (Figures 3(c) and 3(d)). However, Ser473 phosphorylation was not changed in response to Rapamycin treatment in ZL rat hearts (Figures 3(c) and 3(d)). Thus, the increase in cardiac fibrosis in ZL-Rap does not seem to be the effect of Rapamycin-induced Akt Phosphorylation.

### 3.4. Differences in the Intracardiac Cytokine Profile between ZO-C and ZL-C

**Figure 4:**
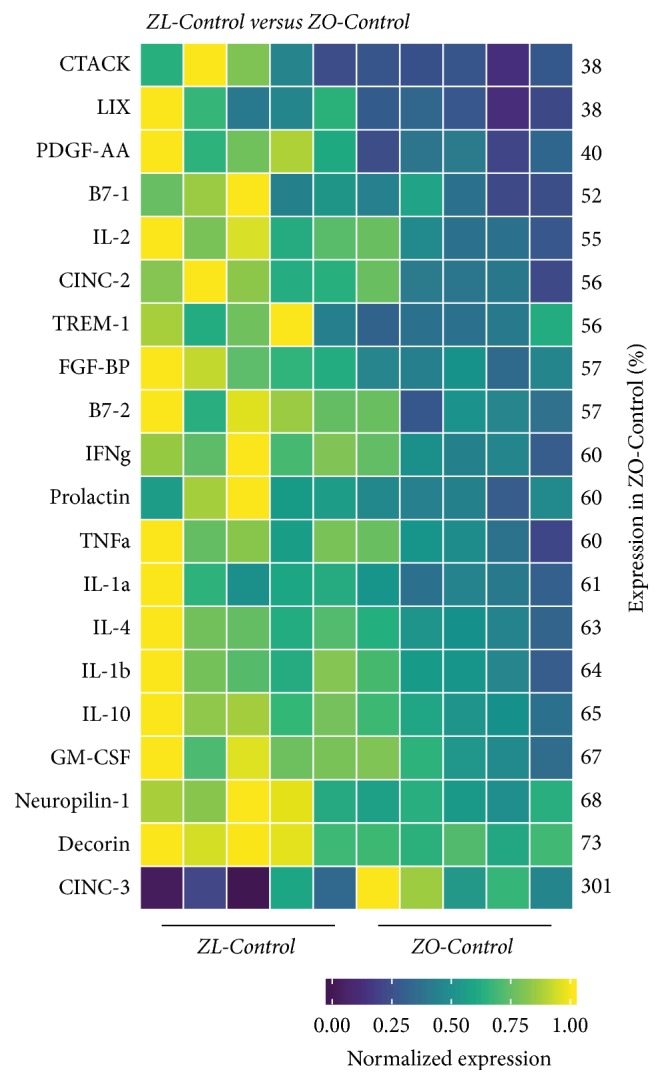
*Changes in cardiac cytokines of ZO-Control rats compared to ZL-Control rats*. Significant differential expression of 20 cytokines was determined in cardiac tissues of ZO-C and ZL-C rats. The heatmap is a graphic representation of relative expression of cardiac protein levels with individual cardiac samples arranged along the *x*-axis and protein markers along the *y*-axis. Expression was normalized for each protein across all animals (across each row). Average relative expression in ZO-C hearts compared to ZL-C hearts for each respective protein is given as a percentage next to each row. Statistical significance was determined using Student's* t*-test. *p* < 0.05 for all proteins, *n* = 5 for each group.

To gain a better understanding of the similarities and differences between the cardiac microenvironment of ZL-C and ZO-C, we examined expression patterns of 67 cardiac proteins using the Rat Cytokine Array Q67 (RayBiotech). Out of 67 proteins, 20 proteins were differentially expressed between ZO-C and ZL-C (*p* < 0.05) (Figure 4). Expression of 19 proteins was suppressed and only one was increased in ZO-C compared to ZL-C. Interestingly, proteins that were suppressed in ZO-C heart included interleukins that are implicated in heart disease. Increased expression of IL-1 alpha and IL-1 beta is associated with poor prognosis for heart failure and suppression of IL-1 beta is reported to reduce leukocyte production and inflammation after acute myocardial infarction [25, 26]. IL-4 is implicated in cardiac fibrosis [27]. These interleukins were suppressed in the heart tissues of ZO-C compared to ZL-C (Figure 4).

However, cardioprotective interleukins (IL-2 and IL-10 [28, 29] that promote regulatory T cell expansion and reduce leukocyte infiltration, resp.) were also suppressed in the ZO-C heart. GM-CSF that primes inflammatory dendritic cell formation and the dendritic cell maturation markers B-71/CD80 and B7-2/CD86 [30] were additionally suppressed in the ZO-C heart. Other proinflammatory cytokines that were suppressed in the ZO-C heart included tumor necrosis factor *α* [31], CTACK (CCL27) [32], and the nursing hormone prolactin that is implicated in heart disease [33]; see Figure 4. Moreover, TREM-1 (that mediates inflammatory cardiac injury) [34], CINC2 (CXCL-3) that is induced by IL-1 (and also called macrophage inflammatory protein 2 beta, MIP-2*β*) [35], and fibroblast growth factor binding protein (FGF-BP) (that is implicated in hypertension) [36] were also suppressed in ZO-C heart (Figure 4).

Several anti-inflammatory markers were also suppressed in ZO-C hearts. These include IL-10 and decorin that ameliorates high fat diet-induced cardiac dysfunction [29, 37], neuropilin that attenuates cardiomyopathy [38], and LIX (CXCL-5) that has a protective role in coronary artery disease [39]; see Figure 4. Other suppressed anti-inflammatory markers in the hearts of ZO-C include interferon-*γ* (IFN-*γ*) that has cardioprotective roles and Platelet Derived Growth Factor (PDGF AA) that improves wound healing [40, 41]. Moreover, the inflammatory cytokine CINC3 (CXCL-2 or macrophage inflammatory protein 2 [MIP-2]) was significantly increased (2-fold) in ZO-C heart (Figure 4) [35]. Thus the intracardiac cytokine profile of ZO-C was considerably different in terms both inflammatory and anti-inflammatory molecule levels compared to ZL-C.

### 3.5. Effect of Rapamycin Treatment on the Intracardiac Cytokines of ZO-C

**Figure 5:**
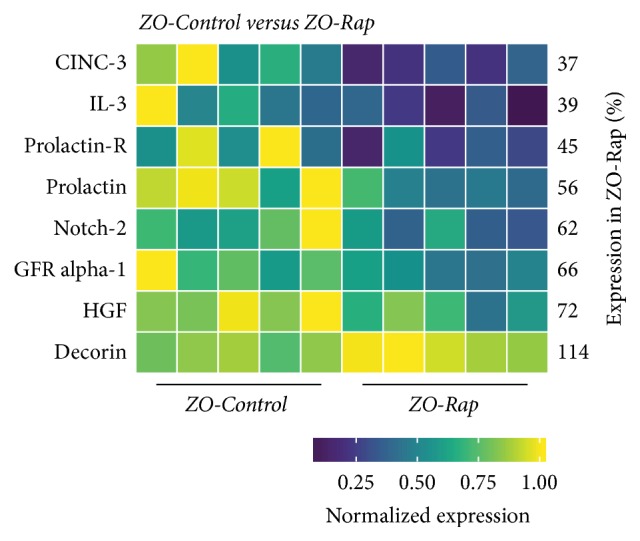
*Changes in cardiac cytokines of ZO-Control rats compared to ZO-Rap rats*. Significant differential expression of 8 cytokines was determined in cardiac tissues of ZO-C and ZO-Rap rats. The heatmap is a graphic representation of relative expression of cardiac protein levels with individual cardiac samples arranged along the *x*-axis and protein markers along the *y*-axis. Expression was normalized for each protein across all animals (across each row). Average relative expression in ZO-Rap hearts compared to ZO-C hearts for each respective protein is given as a percentage next to each row. Statistical significance was determined using Student's* t*-test. *p* < 0.05 for all proteins, *n* = 5 for each group.

**Figure 6:**
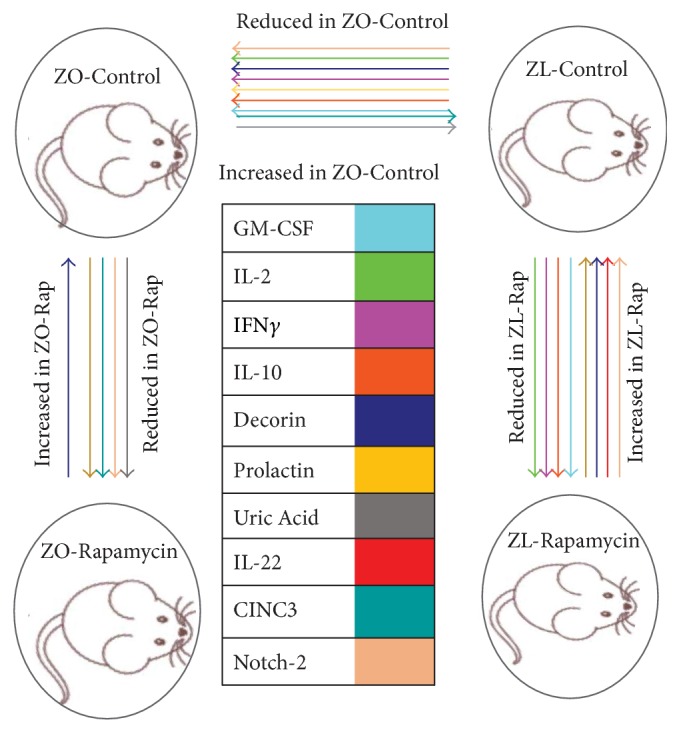
*Rapamycin treatment widened the differences in intracardiac cytokine profiles of ZL-C and ZO-C*. Cartoon diagram shows the intracardiac proteins that were differentially expressed in response to both diabetes and Rapamycin treatment. Arrowhead points towards reduced expression. Direction of change in the protein is labeled. Expressions of GM-CSF, IL-2, IFN-*γ*, and IL-10 are reduced by both diabetes (in ZO-C) and Rapamycin treatment (in ZL-Rap) compared to ZL-C. Prolactin and Notch 2 are suppressed by Rapamycin treatment in ZO rat but increased in response to Rapamycin treatment in ZL rats. Uric acid and CINC-3 were increased by diabetes in ZO rats compared to ZL rats but suppressed by Rapamycin treatment. Decorin was suppressed by diabetes (ZL-C versus ZO-C) but increased by Rapamycin treatment in both ZL and ZO rats. Rapamycin treatment increased IL-22 only in ZL rats. IL-1*α*, IL-1*β*, B7-1 (CD-80), B7-2 (CD-86), and other molecules that were suppressed by diabetes but not modulated by Rapamycin are not shown here.

Eight intracardiac proteins were differentially expressed between ZO-C and ZO-Rap (Figure 5). Out of the 20 intracardiac proteins that were differentially expressed between ZL-C and ZO-C, only the expression of three proteins was modulated by Rap treatment. They were prolactin, decorin, and CINC2/CXCL-3/MIP-2b (Figures 5 and 6). Decorin was suppressed by 28% in ZO-C. Rapamycin treatment partially restored cardioprotective decorin in ZO-C by 14%. Prolactin was also suppressed in ZO-C compared to ZL-C by 40% and was further suppressed by Rapamycin treatment (44%) (Figures 5 and 6) that resulted in a cumulative suppression of prolactin levels by 64% in ZO-Rap compared to healthy ZL-C. CINC3 (CXCL-2/MIP-2) was increased by 2-fold in ZO-C, but Rapamycin treatment suppressed it by 64%. Rapamycin treatment reversed the increase in this inflammatory marker in ZO-C (Figures 5 and 6).

The other five intracardiac proteins that were modulated by Rapamycin treatment in ZO-C were Notch-2, GDNF family receptor alpha-1 (GFR*α*1), hepatocyte growth factor (HGF), IL-3, and prolactin receptor (Figure 5). Mutations in Notch-2 gene that results in deficiency of Notch-2 signaling are associated with congenital heart defects including right-sided obstructive lesions such as pulmonary artery stenosis and tetralogy of Fallot, as well as ventricular septal defects [42]. Therefore, Notch-2 signaling is critical for cardiac structure and loss of Notch-2 signaling via Rapamycin treatment may have detrimental effects in the ZO-C heart. HGF is another important cardioprotective protein [43]. HGF is an angiogenic and antiapoptotic protein that ameliorates cardiac ischemia-reperfusion injury and blockade of endogenous HGF increases infarct size and mortality. Loss of HGF via Rapamycin treatment may also be detrimental to cardiac functions in ZO-C. Conversely, IL-3 is considered as an inflammatory cytokine that is implicated in atherogenesis [44] and prolactin receptor is implicated in the pathology of coronary artery plaques [45]. Therefore, suppression of their expression by Rapamycin treatment may be beneficial for the ZO-C heart.

### 3.6. Effect of Rapamycin Treatment on the Intracardiac Cytokines of ZL-C

**Figure 7:**
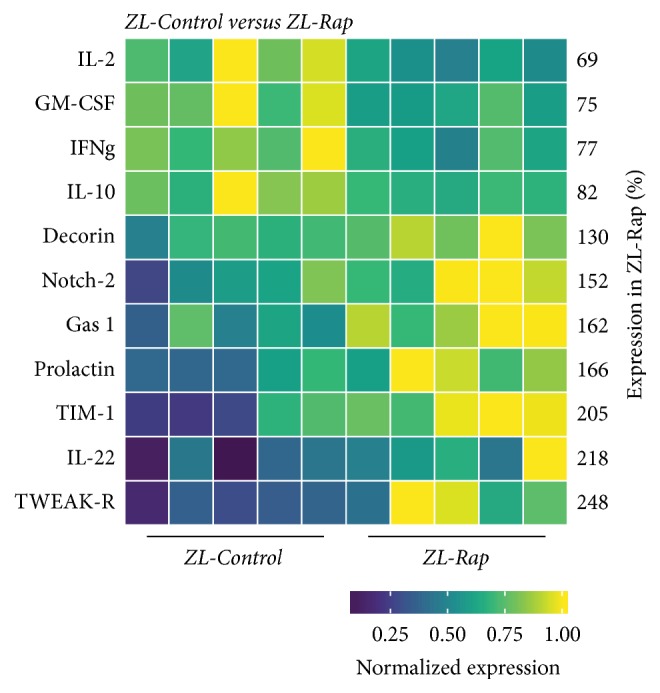
*Changes in cardiac cytokines of ZL-Control rats compared to ZL-Rap rats*. Significant differential expression of 11 cytokines was determined in cardiac tissues of ZL-C and ZL-Rap rats. The heatmap is a graphic representation of relative expression of cardiac protein levels with individual cardiac samples arranged along the *x*-axis and protein markers along the *y*-axis. Expression was normalized for each protein across all animals (across each row). Average relative expression in ZL-Rap hearts compared to ZL-C hearts for each respective protein is given as a percentage next to each row. Statistical significance was determined using Student's* t*-test. *p* < 0.05 for all proteins, *n* = 5 for each group.

There were eleven intracardiac proteins that were differentially expressed between ZL-C and ZL-Rap groups (Figure 7). Expressions of four of these proteins, GM-CSF, IL-2, IL-10, and interferon-*γ*, were suppressed by Rapamycin treatment in ZL-C (Figures 6 and 7). Conversely, while Rapamycin treatment suppressed prolactin and Notch-2 in ZO-C hearts, it increased their expression in ZL-C hearts (Figures 6 and 7). Decorin was another protein whose cardiac expression was modulated similarly in both ZO-C and ZL-C. Rapamycin increased this cardioprotective protein in both groups (Figure 6). Expression of profibrotic receptor for tumor necrosis factor-like weak inducer of apoptosis (TWEAK-R/Fn4) [46] was increased by over 2.4-fold by Rapamycin in the ZL-C heart. The T cell Ig and mucin domain-1 (Tim-1) protein is implicated in allograft rejection [47] and its expression also was increased by 2-fold by Rapamycin treatment. Finally, Rapamycin treatment upregulated the expression of growth arrest specific gene 1 (Gas1) that has antiapoptotic properties [48]. While there were some similarities in the changes in intracardiac protein expression profiles induced by Rapamycin in ZL-C and ZO-C, there were also considerable differences in the proteins and their direction of change (increased versus decreased as seen in prolactin or Notch-2) (Figure 6).

## 4. Discussion

Data presented here shows for the first time that Rapamycin modulates several intracardiac proteins differentially in healthy Zucker lean rats and diabetic Zucker obese rats. Our expectation was that since Rapamycin is an immunosuppressant, it would suppress inflammatory cytokines in the heart of both lean and obese rats to improve their cardiac function. Previous studies have shown that short-term treatment with Rapamycin improves cardiac functions in murine models of diabetes and reduces oxidative stress [14, 15]. Data presented here also show that Rapamycin treatment for six weeks improves many of the cardiac parameters for diastolic dysfunction significantly in both healthy and obese/diabetic rats (Table 1 and Figure 2). However, continuation of the treatment for another six weeks reversed these cardiac functional improvements in ZO rats. Moreover, in healthy ZL-M, Rap treatment caused an increase in cardiac fibrosis. There are no reports in the previous literature of rebound effect of mTORC1 signaling and upregulation due to chronic suppression in animals or humans. Therefore, we excluded that possibility as a causal factor in these results.

In healthy mice, it was shown that an “intermittent rapamycin dosing schedule” could reduce effects on glucose tolerance and the immune system compared to daily rapamycin treatment [49]. However, similar studies are not reported in rodent models with preexisting conditions such as obesity and DM. In another study, 24-month-old female C57BL/6J mice were treated with Rap (microencapsulated Rapamycin diet containing 14 parts per million Rap) for 3 months and it was observed that Rap treatment reversed age-related cardiac dysfunction [50]. In this study, glucose levels were slightly increased in aged healthy mice in response to Rap treatment initially; however, they became similar at the end of the treatment [50]. In our study rats were treated from the age of 2 to 5 months. Thus, the ZL rats (healthy rats) used in our study were much younger and expected to be healthier in terms of metabolic profile, cardiac function, and structure, compared to aged rodent models. We also did not observe any significant changes in glucose levels in our young healthy ZL rats in response to Rap treatment. Authors noted that 45 intracardiac cytokines (out of a total of 145 cytokines tested) were suppressed in Rap-treated aged mice and concluded that “the anti-inflammatory effects of rapamycin are more potent within cardiac tissue than systemically in the sera” [50]. Data presented here also suggest that Rap suppressed intracardiac cytokines in young healthy ZL rats. Interestingly, IL-2, IL-10, and TWEAK-R were among the intracardiac cytokines suppressed by Rap in both young ZL rats and aged mice. There was no indication of any change in the cardiac fibrosis levels in response to Rap treatment in these aged healthy mice [50]. It is conceivable that the aged mice had some cardiac fibrosis already and that was not altered by Rap treatment. However, in young ZL rats, we observed an increase in fibrosis in response to Rap treatment. Moreover, we observed opposing effects of Rap treatment on young ZL and ZO. In ZO, 750 *μ*g/kg/day of Rap treatment was sufficient to suppress cardiac fibrosis, whereas the same treatment increased cardiac fibrosis in ZL. Therefore, many factors including differences the age (young or old), species, route of drug delivery, and dosage seem to influence effects of Rap in rodent models.

Importantly, ZO-Rap exhibited worsening of relative wall thickness,* Vp*,* E*/*Vp*, and isovolumic relaxation time at the end of treatment (12 weeks) compared to the middle of treatment (6 weeks) (Figures 2(b), 2(d), 2(e), and 2(f)) and compared to the untreated ZO-C. Consistent with previous reports on diabetic rats, ZO-C heart exhibited increases in phosphorylation of Ser473 of Akt that results in Akt activation, a contributor to cardiac fibrosis, and enhanced cardiac fibrosis (Figure 3). Rapamycin suppressed both cardiac fibrosis and excessive phosphorylation of Ser473 of Akt (Figure 3). Therefore, the worsening of cardiac functions in ZO-Rap is not related to increased phosphorylation of Ser473 residue of Akt and subsequent increase in fibrosis. However, unlike in healthy mice [50] or ZL rats subjected to 3-month Rap treatment (this study), there was a consistent rise in fasting plasma glucose in ZO-Rap throughout the 3-month period of Rap treatment. Thus ZO-Rap had the highest hyperglycemia among the four groups tested in this study at the end of treatment. It has been proposed that hyperglycemia-mediated activation of nonoxidative glucose pathways (NOGPs), particularly the advanced glycation end-products (AGE) pathway, could have a crucial role in causing dysfunction of cardiac cells [51]. The fasting plasma glucose levels of ZO-Rap was about 12 mmol/L higher than that of ZO-C at the end of treatment. This significant disparity in fasting plasma glucose levels between ZO-C and ZO-Rap at the end of 12-week Rap treatment could have contributed to increased activation of NOGPs in ZO-Rap that independently contributed to worsening of cardiac functions in ZO-Rap compared to ZO-C. Moreover, fasting plasma glucose levels of ZO-Rap after 12-week Rap treatment was about 6 mmol/L higher than that of ZO-Rap after 6-week Rap treatment. This increase in hyperglycemia after 12-week Rap treatment could have also contributed to the reversal of Rap-mediated improvements in cardiac functions of ZO-Rap after 6-week treatment. In ZL-Rap,* Vp* and IVRT were comparable at both time points and* E*/*E*′ further improved by long-term Rapamycin treatment compared to the untreated ZL-C (Figures 2(c), 2(e), and 2(f)). However, ZL-Rap also showed a trend towards increase in relative wall thickness and reduction in* E*/*Vp *(Figures 2(b) and 2(d)).

In ZL rats, Rapamycin treatment suppressed GM-CSF that primes inflammatory dendritic cell formation [30] and improved expression of cardioprotective decorin [37], anti-inflammatory/regenerative IL-22 [52], and antiapoptotic GAS1 [48]. However, it also increased the expression of potential cardiodeleterious molecules such as TWEAK-R/Fn4 [46], Tim1 [47], and prolactin [33]. Moreover, Rapamycin suppressed IL-2, IL-10, and IFN-*γ* [28, 29, 40] which are cardioprotective. There seems to be a shift towards significantly disturbing the cytokine profile seen in healthy animals by long-term Rapamycin treatment.

Interestingly, three antifibrotic cytokines, IL-10 [53, 54], IFN-*γ* [55, 56], and GM-CSF [57], were suppressed in ZO-C and ZL-Rap heart compared to ZL-C (Figures 4, 6, and 7). Because both ZO-C and ZL-Rap hearts exhibited fibrosis, it is conceivable that suppression of these antifibrotic cytokines either by DM or by Rapamycin serves as an additional mechanism for fibrosis. These molecules were not significantly suppressed by Rapamycin in the ZO-Rap heart (Figure 5). Thus, the effect of Rapamycin on these antifibrotic cytokines differed in ZL and ZO hearts. This differential effect of Rapamycin on antifibrotic cytokines in healthy and diabetic rats could have contributed to Rapamycin-induced fibrosis in healthy ZL heart and suppression of fibrosis in diabetic ZO heart.

**Figure 8:**
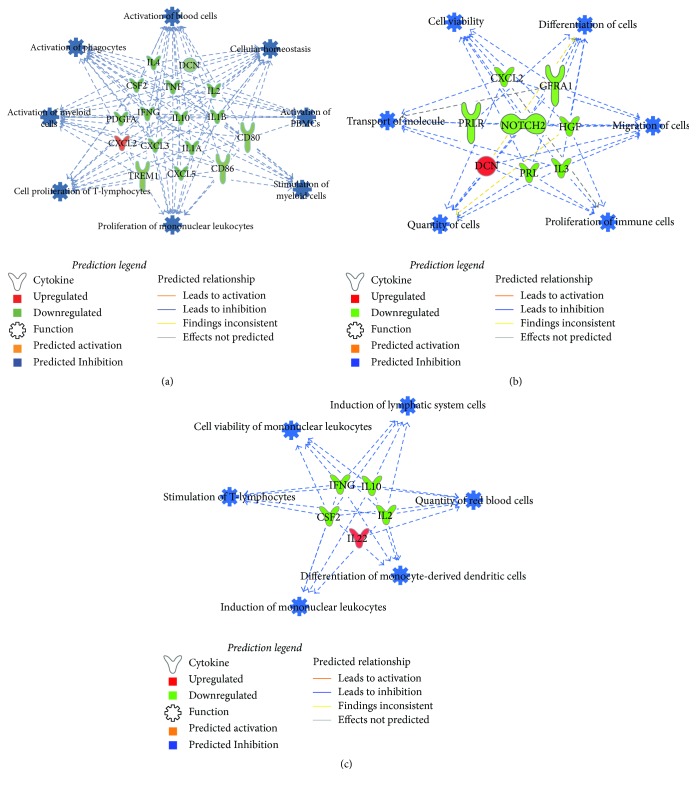
Predicted intracardiac immune responses modulated by DM or Rapamycin treatment or their combination. Disease and function networks generated by bioinformatic pathway analysis through the use of the Ingenuity Pathway Analysis (IPA®, QIAGEN Redwood City) software (65) for ZO-C versus ZL-C (a), ZO-Rap versus ZO-C (b), and ZL-Rap versus ZL-C (c) are shown. Ensemble ID numbers for the genes encoding proteins listed in Figures 4, 5, and 7 were used as input for IPA. List of gene symbols used in networks and respective proteins is given in (d). Predicted functions/diseases with the highest absolute activation* z*-scores were combined into networks for each comparison. Direction of change in expression is indicated by colors of the molecules as indicated in the legend shown. IPA analysis indicated suppression of various immune functions (shown in blue as described in the legend) in the heart tissues of ZO-C compared to ZL-C, ZO-Rap compared to ZO-C, and ZL-Rap compared to ZL-C.

Obesity and diabetes are considered states of chronic inflammation and are characterized by an impaired immune response and increased risk for infections [58, 59]. It was interesting to note that there was significant suppression in the expression of both inflammatory and anti-inflammatory interleukins in the ZO-C heart compared to ZL-C heart. To understand how this change in cytokine profile modulates disease, we used Ingenuity Pathway Analysis (IPA) of the differentially expressed cytokines between ZL-C and ZO-C.  Figure 8(a) shows that IPA diseases and functions analysis predicts suppression of many key immune processes including activation of phagocytes, myeloid cells, blood cells, and peripheral blood mononuclear cells (PBMCs) in ZO-C heart compared to ZL-C heart. Moreover, several inflammatory molecules that are critical for immune response to infections were suppressed significantly in ZO-C heart compared to ZL-C heart. These include IL-1 alpha and IL-1 beta, IL-2, GM-CSF (that primes inflammatory dendritic cell formation), the dendritic cell maturation markers B7-1/CD80 and B7-2/CD86, CTACK/CCL27 (that plays a role in priming T_reg_ cells) [60], and TREM-1 that promotes host defense during early stage of infection [61]. The only proinflammatory cytokine that was increased in ZO-C heart compared to ZL-C heart was CINC-3 (CXCL2/MIP-2). These observations indicate the heart tissues of ZO-C rats may have a comparatively weaker host defense compared to the heart tissues of ZL-C rats. It must be noted that endocarditis and skin and soft tissue infections are higher in diabetics [58, 62]. It is conceivable that suppression of cytokines essential for intracardiac immune functions in diabetes may contribute to this pathology.

Rapamycin treatment reversed suppression of cardioprotective decorin in ZO rat but did not recover expression of other molecules including cardioprotective IL-10. It also further suppressed prolactin and also prolactin receptor. Thus, potential inflammatory signaling from prolactin was significantly suppressed in ZO rat heart after Rapamycin treatment. Rapamycin treatment also reversed the increase in CINC-3 (CXCL2/MIP-2) in ZO rats. These effects are potentially cardioprotective. However, it suppressed cardioprotective HGF and also Notch-2 that is implicated in adult cardiac repair [43, 63] in ZO-C heart. Therefore, while Rapamycin treatment had a beneficial effect in restoring the expression of some of the intracardiac molecules to the levels seen in the healthy ZL rat, its effects were mixed as it also contributed to further weakening of the cardiac health by suppressing additional cardioprotective molecules.

IPA analysis of the differentially expressed cytokines between ZL-C and ZL-Rap predicted suppression of immune functions including quantity of red blood cells, differentiation of monocyte derived dendritic cells, and cell viability, and induction of mononuclear leukocytes (Figure 8(b)). This is not surprising since Rapamycin is an immunosuppressant. Similarly, IPA analysis of the differentially expressed cytokines between ZO-C and ZO-Rap also predicted suppression of key cellular functions including quantity of cells, differentiation of cells, migration of cells, and proliferation of immune cells. These Rapamycin-induced suppression of cellular functions in ZO rats predicted by IPA is in addition to the suppression of many key immune functions as shown in Figure 8(a). Thus, the cytokine profile of ZO-Rap indicates additional weakening of intracardiac innate immune functions compared to ZO-C resulting from a pan-suppression of cellular functions by Rapamycin.

**Figure 9:**
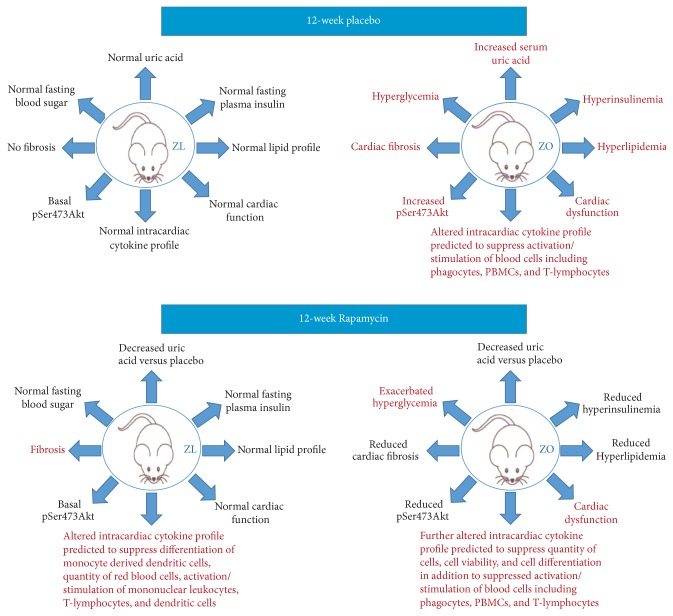
Summary of the effects of DM and Rapamycin treatment on metabolic and cardiac outcomes in diabetic and healthy rats.

Effect of Rapamycin treatment on cardiac dysfunction in diabetic murine models has been studied extensively for short time periods and the results are encouraging for the use of Rapamycin in diabetics. Our data is consistent with previous reports that show Rapamycin reduced cardiac fibrosis and hyperinsulinemia in diabetic murine models. However, Rapamycin-induced cardiac fibrosis in healthy ZO heart and suppressed anti-inflammatory cytokines (IL-10, IFN*γ*, and GM-CSF) only in ZL heart but not in ZO heart. These observations highlight the potential role of differential modulation of intracardiac cytokine profile by Rapamycin in health and diabetes in shaping the cardiac outcome to the treatment. To our knowledge, there are no reports that define intracardiac cytokine profile in diabetic murine models and compare the effect of Rapamycin treatment on the intracardiac cytokine profiles of healthy and diabetic rats. In Figure 9, we have summarized how metabolic and cardiac parameters of ZL-C and ZO-C differ in the absence of and after Rapamycin treatment. One notable factor here is the altered cytokine profile of the ZO-C heart compared to ZL-C heart that resulted in IPA predicted suppression of intracardiac immune response in ZO-C. This interpretation is consistent with the idea that tissue immune response is impaired in diabetics and this condition can render diabetics to increased susceptibility to infections. Since Rapamycin is an immunosuppressant, in diabetics with an impaired immune response, use of Rapamycin that can further increase immune suppression requires additional caution.

## 5. Conclusion

In summary, data presented here shows for the first time that diabetic ZO-C rats have an intracardiac cytokine protein expression profile that is reflective of a weaker cardiac host defense compared to that seen in the healthy ZL-C rat. Rapamycin treatment, over the short term (6 weeks), improved several cardiac parameters of diastolic dysfunction in ZO-C. It also suppressed elevation in triglycerides and insulin in the blood throughout the 12-week treatment period. Moreover, elevation in serum uric acid levels, a surrogate marker for heart disease, was suppressed to comparable levels seen in ZL-C at the end of Rapamycin treatment. These observations support the concept that Rapamycin has metabolic and cardiac protective effects in diabetic ZO rats. However, hyperglycemia was further exacerbated by Rapamycin treatment in ZO rats and this is consistent with suppression of insulin. Long-term treatment with Rapamycin (12 weeks) reversed or attenuated improvements in cardiac functional parameters that were observed after six-week treatment and exacerbated the disease. This effect could be due to the fact that DM became more severe in ZO-Rap and the significant change in the microenvironment of the heart where several cardioprotective and host defense molecules including IL-10 and HGF are reduced could have influenced the differential outcome of Rapamycin intervention on the cardiac functions of ZL-C and ZO-C. Importantly, our data on the intracardiac cytokine protein profiling indicate a significant weakening of intracardiac immune defense in ZO-C compared to ZL-C. Rapamycin is an immunosuppressant and seems to have contributed to suppression of immunity in the background of an already weakened immune response in ZO-C. It is conceivable that the fundamental difference in the intracardiac cytokine profile of the ZL-C and ZO-C is an important contributing factor to the differential cardiac outcomes in diabetics and nondiabetics in response to Rapamycin treatment.

We have shown previously that Rapamycin treatment suppresses Myeloid Cell Leukemia 1 (MCL-1) that has a critical role in cardiomyocyte and vascular smooth muscle survival [17]. We have reported recently that a novel peptide agonist for the angiotensin II type 2 receptor AT2R named NP-6A4 is effective in improving cardiomyocyte and vascular smooth muscle survival under nutrient deficiency stress [64]. We also showed that NP-6A4 could increase MCL-1 expression in cardiovascular cells [64]. We propose that a synergistic treatment with Rapamycin and NP-6A4 would be beneficial in mitigating some of the negative effects of Rapamycin treatment and enhancing cardioprotection in diabetes.

## Figures and Tables

**Table 1:** Summary of 2D M-Mode, pulse wave, and tissue Doppler echo measurements on 14-week-old ZL-C and ZO-C and Rapamycin treated (ZL-Rap and ZO-Rap) rats. Values are mean ± SE. Numbers in parentheses are sample sizes. ^*∗*^*p* < 0.05 ZL-C versus ZO-C; ^†^*p* < 0.05 ZO-C versus ZO-Rap; ^‡^*p* < 0.05 ZL-C versus ZL-Rap; ^§^*p* < 0.05 ZL-Rap versus ZO-Rap.

Parameter	Main effect	*p* value	ZL-C (4)	ZL-Rap (4)	ZO-C (4)	ZO-Rap (4)
PWTd, cm	Strain	**0.001**	0.15 ± 0.01	0.15 ± 0.00	0.20 ± 0.01^*∗*†^	0.17 ± 0.01
	Treatment	**0.036**				
	Interaction	**0.036**				

RWT	Strain	**0.050**	0.41 ± 0.02	0.45 ± 0.01	0.53 ± 0.05^*∗*^	0.48 ± 0.04
	Treatment	0.842				
	Interaction	0.257				

LA, cm	Strain	**0.018**	0.27 ± 0.03	0.28 ± 0.04	0.40 ± 0.02^*∗*†^	0.31 ± 0.01
	Treatment	0.162				
	Interaction	0.090				

*E*, m·s^−1^	Strain	**0.016**	1.09 ± 0.05	1.11 ± 0.06	1.26 ± 0.05^*∗*^	1.21 ± 0.03
	Treatment	0.750				
	Interaction	0.480				

*E*/*E*′	Strain	**0.015**	15.3 ± 0.7	13.8 ± 1.06	22.2 ± 0.9^*∗*†^	15.6 ± 2.6
	Treatment	**0.021**				
	Interaction	0.119				

*E*′/*A*′	Strain	**0.001**	1.21 ± 0.02	1.15 ± 0.08	0.86 ± 0.05^*∗*†^	1.08 ± 0.06
	Treatment	**0.014**				
	Interaction	0.542				

*Vp*	Strain	**0.037**	57 ± 3	67 ± 2^a^	47 ± 1^a†^	61 ± 6
	Treatment	**0.005**				
	Interaction	0.567				

*E*/*Vp*	Strain	**0.001**	1.92 ± 0.17	1.65 ± 0.05	2.67 ± 0.11^*∗*†^	2.03 ± 0.18
	Treatment	**0.006**				
	Interaction	0.200				

IVRT	Strain	**0.002**	17.8 ± 1.5	16.1 ± 1.3	23.3 ± 1.9^*∗*^	21.8 ± 0.8^§^
	Treatment	0.301				
	Interaction	0.966				

MPI	Strain	**0.011**	0.38 ± 0.01	0.34 ± 0.01	0.45 ± 0.02^*∗*†^	0.38 ± 0.02
	Treatment	**0.016**				
	Interaction	0.373				

PWTd, posterior wall thickness-diastole; RWT, relative wall thickness; LA, left atrial diameter; *E*, velocity of early mitral flow; *E*′, peak velocity of septal annulus; *E*/*E*′ index of LA filling pressure; *Vp*, flow propagation velocity; *E*/*Vp*, index of LV filling pressure; IVRT, isovolumic relaxation time; MPI, myocardial performance index. ^a^*P* = 0.06 versus ZL-C.

**Table 2:** Summary of 2D M-Mode, pulse wave, and tissue Doppler echo measurements on 20-week-old ZL-C and ZO-C and Rapamycin-treated (ZL-Rap and ZO-Rap) rats. Values are mean ± SE. Numbers in parentheses are sample sizes. ^*∗*^*p* < 0.05 ZL-C versus ZO-C; ^†^*p* < 0.05 ZO-C versus ZO-Rap; ^‡^*p* < 0.05 ZL-C versus ZL-Rap; ^§^*p* < 0.05 ZL-Rap versus ZO-Rap.

Parameter	Main effect	*p* value	ZL-C (4)	ZL-R (4)	ZO-C (4)	ZO-R (4)
HR	Strain	**0.019**	360 ± 16	389 ± 12	349 ± 10	339 ± 9^§^
	Treatment	0.441				
	Interaction	0.114				

SWTd, cm	Strain	**0.001**	0.15 ± 0.00	0.15 ± 0.00	0.17 ± 0.01^*∗*^	0.19 ± 0.01^§^
	Treatment	0.147				
	Interaction	0.308				

PWTd, cm	Strain	**0.004**	0.16 ± 0.00	0.17 ± 0.00	0.19 ± 0.01^*∗*^	0.20 ± 0.01^§^
	Treatment	0.116				
	Interaction	0.734				

LVIDd, cm	Strain	0.757	0.73 ± 0.02	0.67 ± 0.02^a^	0.74 ± 0.01^†^	**0.65 ± 0.03**
	Treatment	**0.003**				
	Interaction	0.597				

RWT	Strain	**0.001**	0.42 ± 0.01	0.48 ± 0.02^‡^	0.48 ± 0.02^*∗*†^	0.60 ± 0.03^§^
	Treatment	**0.001**				
	Interaction	0.163				

LA, cm	Strain	**0.040**	0.28 ± 0.01	0.31 ± 0.03	0.40 ± 0.03^*∗*†^	**0.29 ± 0.03**
	Treatment	0.135				
	Interaction	**0.017**				

*E*′, m·s^−1^	Strain	**0.001**	0.078 ± 0.014	0.123 ± 0.009^‡^	0.060 ± 0.007	0.056 ± 0.006^§^
	Treatment	**0.037**				
	Interaction	**0.016**				

*E*/*E*′	Strain	**0.013**	16.2 ± 3.3	9.5 ± 1.2	19.8 ± 3.7	21.2 ± 2.2^§^
	Treatment	0.340				
	Interaction	0.152				

*E*′/*A*′	Strain	**0.016**	1.20 ± 0.25	2.48 ± 0.32^‡^	1.09 ± 0.18	1.14 ± 0.26^§^
	Treatment	**0.025**				
	Interaction	**0.036**				

*Vp*	Strain	**0.011**	65 ± 6	63 ± 4	53 ± 7	43 ± 3^§^
	Treatment	0.256				
	Interaction	0.465				

*E*/*Vp*	Strain	**0.024**	1.78 ± 0.20	1.83 ± 0.08	2.26 ± 0.48	**2.70 ± 0.17**
	Treatment	0.374				
	Interaction	471				

IVRT	Strain	**0.001**	19 ± 1.7	16.7 ± 0.6	26.0 ± 1.1^*∗*^	28.0 ± 1.3^§^
	Treatment	0.919				
	Interaction	0.100				

MPI	Strain	**0.005**	0.41 ± 0.03	0.38 ± 0.01	0.48 ± 0.03^a^	0.48 ± 0.03^§^
	Treatment	0.487				
	Interaction	0.553				

HR, heart rate; SWTd, septal wall thickness-diastole; PWTd, posterior wall thickness-diastole; LVIDd, LV inner dimension-diastole; RWT, relative wall thickness; LA, left atrial diameter; *E*, velocity of early mitral flow; *E*′, peak velocity of septal annulus; *E*/*E*′ index of LA filling pressure; *Vp*, flow propogation velocity; *E*/*Vp*, index of LV filling pressure; IVRT, isovolumic relaxation time; MPI, myocardial performance index.
